# Prognostic implications of metabolism-related genes in acute myeloid leukemia

**DOI:** 10.3389/fgene.2024.1424365

**Published:** 2024-10-03

**Authors:** Na Ren, Jianan Wang, Ruibing Li, Chengliang Yin, Mianyang Li, Chengbin Wang

**Affiliations:** ^1^ Medical School of Chinese PLA, Beijing, China; ^2^ Department of Laboratory Medicine, The First Medical Center of Chinese PLA General Hospital, Beijing, China; ^3^ Department of Laboratory Medical Center, General Hospital of Northern Theater Command, Shenyang, China; ^4^ Medical Innovation Research Division, Chinese PLA General Hospital, Beijing, China

**Keywords:** acute myeloid leukemia, metabolism-related gene, prognostic signature, immune cell infiltration, drug sensitivity, CA13

## Abstract

**Introduction:**

Acute myeloid leukemia(AML) is a diverse malignancy with a prognosis that varies, being especially unfavorable in older patients and those with high-risk characteristics. Metabolic reprogramming has become a significant factor in AML development , presenting new opportunities for prognostic assessment and therapeutic intervention.

**Methods:**

Metabolism-related differentially expressed genes (mDEGs) were identified by integrating KEGG metabolic gene lists with AML gene expression data from GSE63270. Using TCGA data, we performed consensus clustering and survival analysis to investigate the prognostic significance of mDEGs. A metabolic risk model was constructed using LASSO Cox reg ression and enhanced by a nomogram incorporated clinical characteristics. The model was validated through receiver operating characteristic (ROC) curves and survival statistics. Gene network analysis was conducted to identify critical prognostic factors. The tumor immune microenvironment was evaluated using CIBERSORT and ESTIMATE algorithms, followed by correlation analysis between immune checkpoint gene expression and risk scores. Drug sensitivity predictions and *in vitro* assays were performed to explore the effects of mDEGs on cell proliferation and chemoresistance.

**Results:**

An 11-gene metabolic prognostic model was established and validated. High-risk patients had worse overall survival in both training and validation cohorts (*p* < 0.05). The risk score was an independent prognostic factor. High-risk patients showed increased immune cell infiltration and potential response to checkpoint inhibitors but decreased drug sensitivity. The model correlated with sensitivity to drugs such as venetoclax. Carbonic anhydrase 13 (CA13) was identified as a key gene related to prognosis and doxorubicin resistance. Knocking down CA13 reduced proliferation and increased cell death with doxorubicin treatment.

**Conclusion:**

A novel metabolic gene signature was developed to stratify risk and predict prognosis in AML, serving as an independent prognostic factor. CA13 was identified as a potential therapeutic target. This study provides new insights into the prognostic and therapeutic implications of metabolic genes in AML.

## 1 Introduction

Acute myeloid leukemia (AML) is a heterogeneous malignancy of myeloid precursors characterized by uncontrolled proliferation and differentiation impediments of hematopoietic cells, resulting in impaired hematopoiesis and bone marrow failure (Serroukh et al., 2023; Papaemmanuil et al., 2016). As the most common type of acute leukemia in adults, the estimated 5-year overall survival (OS) of AML is about 30% varying significantly across different age groups, approaching 50% in younger patients but less than 10% in patients older than 60 years (Sasaki et al., 2021). In recent years,significant improvements in therapeutic efficacy and outcomes of AML patients have been achieved due to advances in diagnostic and prognostic stratification, improvements in supportive treatment, and resolution of donor sources for allogeneic hematopoietic stem cell transplantation (allo-HSCT) (Wang et al., 2015; Kanakry et al., 2016; Wang et al., 2018; Xu et al., 2018). However, the prognosis for some AML patients, especially the elderly and those with adverse-risk features who are prone to drug resistance, remains relatively poor (Short and Ravandi, 2016). Accumulating evidence indicates that a comprehensive stratified evaluation of prognosis is the basis for premise treatment or relapse intervention for AML patients (Döhner et al., 2017). Risk stratification based on cytogenetics and genomic signatures has been widely used in clinical practice to identify various risk groups. However, current stratification methods have limitations in precisely predicting the outcome of all the AML patients due to the diversity of genetic mutation and high heterogeneity of AML. Therefore, there is an urgent need to explore more risk features that further improve clinical outcome and treatment guidance.

Metabolism reprogramming is an emerging hallmark of cancer cells, playing an important role in the leukemogenesis and AML prognosis. To meet the growing bioenergetic and biosynthetic demands for survival and proliferation, AML cells abnormally regulate fluxes of metabolites through a variety of metabolic pathways, including lipid metabolism and carbohydrate metabolism. Metabolic reprogramming is not only an important manifestation of AML but also clinically relevant to risk stratification and therapeutic targeting (Wojcicki et al., 2020). Carbohydrate metabolism is significantly enhanced in AML cells and the inhibition of glycolysis suppresses the proliferation of leukemia cells and enhances the cytotoxicity of cytarabine (Chen et al., 2014). The therapeutic use of targeted inhibitors showed that prognostic outcomes, including event-free survival (EFS) and OS, are improved in isocitrate dehydrogenase 1 (IDH1)-mutated AML (Döhner et al., 2022; Montesinos et al., 2022). Additionally, abnormity in the lipid metabolism has been found associated with AML prognosis (Wang et al., 2013).

Recently, investigations on the metabolism-related genes (MRG) have shown potential application in the therapeutic targets and prognostic evaluation of AML. Risk models based on metabolism-related genes of carbohydrate, lipid, amino acid and mitochondrion have been proposed, depicting signatures that contribute to better understanding of metabolism-related genes as promising prognostic biomarkers and therapeutic targets for AML (Tong and Zhou, 2023; Yang et al., 2022; Li et al., 2022; Zhou et al., 2022; Zhai et al., 2023). Although these predictive models have suggested the linkage between MRGs and AML prognosis, few have been widely applied, and larger-scale cohorts or experimental verification is still needed. In this study, we integrated transcriptional and clinicopathological data AML cases in The Cancer Genome Atlas (TCGA) database to construct a construct a novel prognostic risk model, and to identify potential prognostic biomarkers and chemotherapy targets for AML with *in vitro* verification. Our aim is to provide novel insights into the metabolism-related prognostic evaluation and therapeutic targets for AML.

## 2 Data and methods

### 2.1 Data source

The gene expression profiles of GSE63270 were obtained from the Gene Expression Omnibus database (http://www.ncbi.nlm.nih.gov/geo/), including samples from 62 AML patients and 42 healthy individuls (Table 1) AML cases from the TCGA database were selected, and their clinical and RNA sequencing data were obtained from the Genomic Data Commons Data Portal (https://portal.gdc.cancer.gov/). Raw counts data of TCGA cohort were converted to counts per million (CPM) expression values using the CPM function in edgeR, and standardized and log2-transformed (log2CPM) with the voom function in the limma. Metabolism-related genes were extracted from the KEGG database (https://www.genome.jp/kegg/pathway.html).

**TABLE 1:** Baseline characteristics of AML patients in three independent cohorts.

Characteristics	TCGA	GSE63270	TARGET
Case No.	151	104	240
Age, n (%)			
<60	88 (58.3)	49 (47.1)	
≥60	63 (41.7)	55 (52.8)	-
Gender, n (%)			
Male	83 (55.0)	-	126
Female	68 (45.0)	-	114
FAB, n (%)			
M0	15 (10.0)	-	30 (12.5)
M1	35 (23.2)	-	23 (9.58)
M2	38 (25.2)		29 (12.08)
M3	15 (10.0)	-	27 (11.25)
M4	29 (19.2)	-	22 (9.17)
M5	15 (10.0)		19 (7.92)
M6	2 (1.2)		31 (12.9)
M7	1 (0.6)	-	30 (12.5)
Unknown	1 (0.6)	-	29 (12.08)
Alive	54 (35.8)	43 (41.3)	129 (53.75)
Dead	97 (64.2)	61 (58.7)	111 (46.25)

### 2.2 Differential expression and enrichment analysis

Differentially expressed genes (DEGs) between AML and healthy individuals in GSE63270 were screened out, |log FC| > 1.5 and false discovery rate (FDR) < 0.05. Differential analysis was performed using the R software version 4.1.0, utilizing the “ggplot2” package (version 3.3.5) for visualization, and the VennDiagram package (version 1.7.3) for generating the Venn diagrams. To explore the biological functions and potential pathways of the mDEGs, gene ontology (GO) Dolinski et al., 2000 (Ashburner et al., 2000) and Kyoto Encyclopedia of Genes and Genomes (KEGG) pathway enrichment analysis (Kanehisa and Goto, 2000) were performed using the cluster Profiler package in R. Functional classifications with a false discovery rate (FDR) less than 0.05 were considered significant.

### 2.3 Unsupervised consensus clustering based on expression profiles

The expression data of the mDEGs were extracted from TCGA, and unsupervised clustering of the mDEGs was performed using the k-means algorithm in Consensus Cluster Plus to identify subtypes. Survival analysis was performed to compare the prognostic difference between the clusters.

### 2.4 Construction and validation of the metabolic risk score

Univariate Cox regression was used to identify prognostic MRGs (*p* < 0.05). A LASSO Cox model was built using the glmnet package (version4.1–1) (Friedman et al., 2010), with the optimal penalty parameter determined by 1000-fold cross-validation. The risk score was calculated as the sum of the product of each gene’s expression and its corresponding coefficient.

TCGA-LAML samples were randomly divided into training and validation sets. The LASSO model was constructed in the training set and validated in the validation set and the entire dataset. ROC analysis was used to evaluate the model’s performance. Patients were stratified into high- and low-risk groups based on the median risk score Table 2. Survival analyses were conducted in the training, validation, and entire sets, as well as an independent TARGET-AML cohort.

**TABLE 2:** Distribution of patients in high and low groups according to clinical factors.

Charactor	TCGA-AML			Charactor TARGET-AML		
Variables	High-risk group	Low-risk group	*p*-value	High-risk group	Low-risk group	*p*-value
Age			0.0217			0.19669
<11 years	34	29		54	65	
>11 years	32	45		66	55	
Gender			0.8614			0.244,688
Female	30	31		62	52	
Male	36	43		58	68	
Status			<0.0001			<0.0001
Alive	14	38		47	82	
Dead	52	36		73	38	
White blood cell			0.2108			1
<10 ¡Á 10^9/L	22	29		54	53	
¡Ý 10 ¡Á 10^9/L	44	45		66	67	
Platelet count			0.6616			0.896,359
<100 ¡Á 10^9/L	54	52		67	69	
¡Ý 100¡Á 10^9/L	12	22		53	51	
Bone marrow blast			0.6779			0.196,317
<70%	52	50		57	68	
¡Ý 70%	14	24		63	52	
Risk (Cytogenetic)			0.0009			2.91E-53
Favorable	6	24		0	120	
Intermediate	43	31		0	0	
Poor	17	19		120	0	
ELN2017			0.0105			0.853,885
Good	9	22		37	40	
Intermediate	34	20		45	41	
Adverse	23	32		38	39	
Chemotherapy			0.0148			0.195,943
Yes	22	10		69	58	

### 2.5 Establishment of nomogram risk prediction model

A prognostic nomogram was constructed using factors identified through univariate Cox regression analysis. The nomogram visualized the score for each variable on a point scale, utilizing the “rms” R package. Predictive probabilities for 1-, 3- and 5-year clinical outcomes were evaluated using calibration curves. Additionally, ROC curves were employed to assess the model’s accuracy.

### 2.6 Survival analysis of risk genes and functional analyses

The single risk gene survival analysis based on OS and the median expression cutoff was performed using Kaplan-Meier (KM) on GEPIA2 (http://gepia2.cancer-pku.cn/) within AML groups. GSEA was performed to compare the enrichment of GO and KEGG pathways between high- and low-risk groups using the clusterProfiler package (*p* < 0.05). FRIEND analysis was conducted using the GOSemSim package to evaluate functional similarity among prognostic genes.

### 2.7 Immune infiltration and immune checkpoint expression profiles

The abundance of 22 immune cell types in TCGA samples was estimated using CIBERSORT (Chen et al., 2018). Differences in immune infiltration between risk groups were compared using the Wilcoxon test. Correlations between immune checkpoint gene expression and risk scores were calculated using Pearson correlation. ESTIMATE was used to quantify the immune score for each sample (Yoshihara et al., 2013).

### 2.8 Drug sensitivity analysis

Based on the preceding analysis, The prophetic R package was used to predict the half-maximal inhibitory concentration (IC50) values for acute myeloid leukemia (AML) patients stratified into high and low-risk categories according to a metabolic gene risk score model, with the median score as the cutoff. IC50 values for various drugs were calculated for each risk group using the predictive algorithms. The Wilcoxon rank-sum test was then applied to evaluate the differences in IC50 values between these groups, facilitating the identification of drugs significantly associated with the risk score.

### 2.9 Cell lines and drug resistance

K562, HL60, and THP1 cell lines were obtained from the PLA Hematology Laboratory (Beijing, China), while K562/A and HL60/A were sourced from the Tianjin Institute of Hematology (Tianjin, China). The THP1/A line was developed in our lab. Doxorubicin (ADR) was sourced from MCE (United States). Cell cultures were maintained at 37°C with 5% CO_2_ in RPMI-1640 medium (Procell, China), supplemented with 10% fetal bovine serum, 100 U/mL penicillin, and 100 μg/mL streptomycin.

Chemoresistant lines (HL60/A, THP1/A, K562/A) were developed by gradually increasing doxorubicin concentrations and selecting resistant cells. For HL60/A, initial ADR concentrations ranged from 0.2–0.4 μg/mL, with a final concentration of 0.5 μg/mL. For K562/A, initial concentrations were 0.6–0.8 μg/mL, with a final concentration of 1 μg/mL. For THP1/A, initial concentrations were 1.0–1.2 μg/ML, with a final concentration of 1.5 μg/mL. One week before experiments, cells were cultured in a doxorubicin-free medium to remove residual drug effects.

### 2.10 *CA13* correlated with chemoresistance and cell proliferation

Based on previous analyses, *CA13* was selected for further *in vitro* verification. *CA13*-targeted small interfering RNA (siRNA) and a negative control siRNA (NC) were designed and synthesized by GenePharma (Suzhou, China). A mixture of three specific siRNA oligos targeting different sites was designated as si-*CA13*. Transfection was performed using Lipofectamine 3,000 (Invitrogen, Thermo Fisher, United States) according to the manufacturer’s protocol. Quantitative PCR (qPCR) was employed to confirm CA13 expression levels. The primer and siRNA sequences are provided in Supplemental Table 1.

To assess the impact of CA13 knockdown on cell proliferation and doxorubicin (DOX) resistance, a CCK-8 assay was conducted to evaluate leukemia cell survival following treatment with 10 μmol/L DOX. Growth curves were utilized to compare survival rates between the si-NC and si-CA13 groups.

### 2.11 Statistical analysis

GraphPad Prism 10 (GraphPad Software, United States) was used for statistical analysis of laboratory data. Comparisons between groups were performed with appropriate statistical tests based on the distribution of the variables. Two-way ANOVA was used for comparison of growth curves. A two-tailed *p* < 0.05 was considered statistically significant.

**FIGURE 1:**
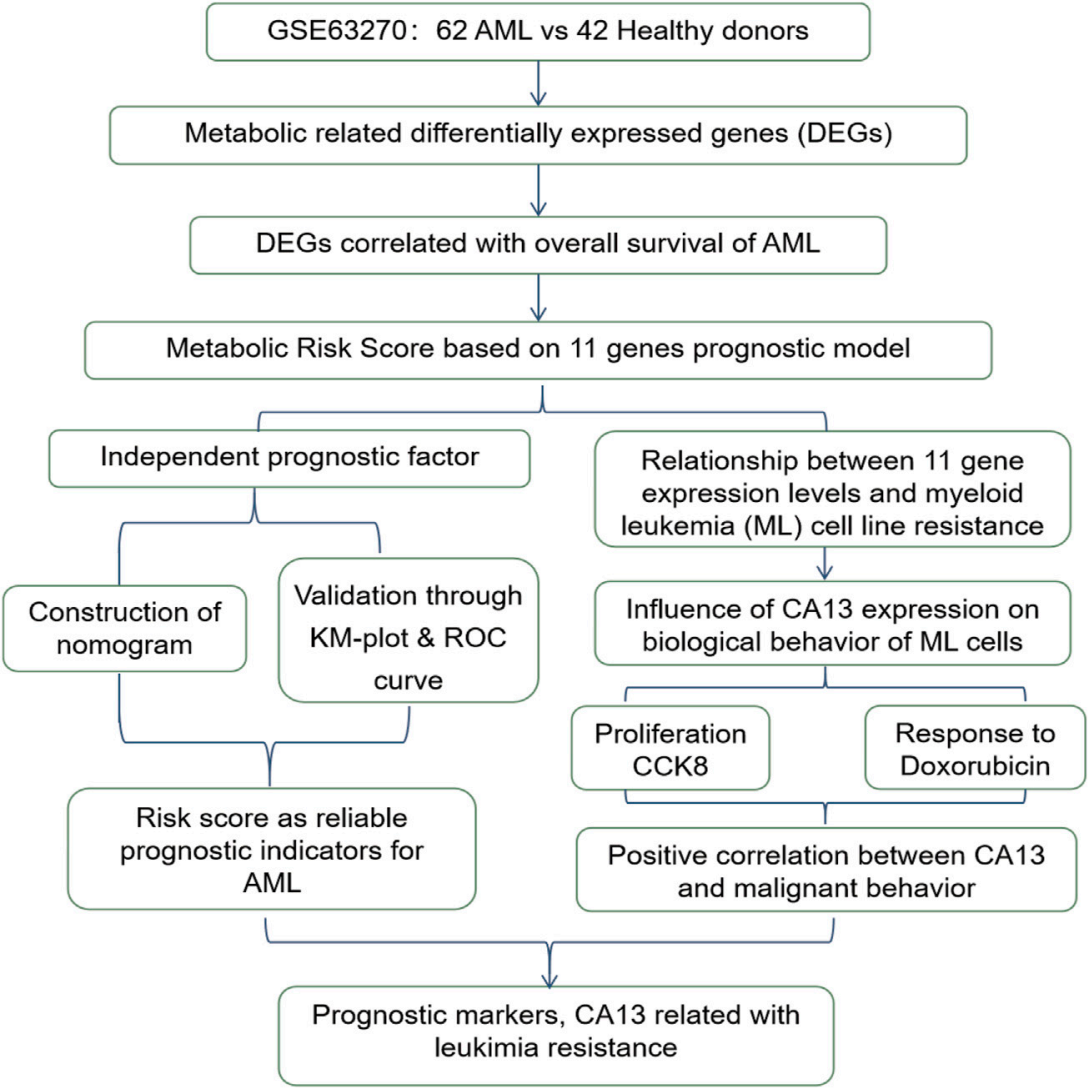
The workflow chart of the study design and analysis.

The total study design is illustrated in Figure 1.

## 3 Results

### 3.1 Identification of metabolism-related differentially expressed genes (mDEGs) in AML

**FIGURE 2:**
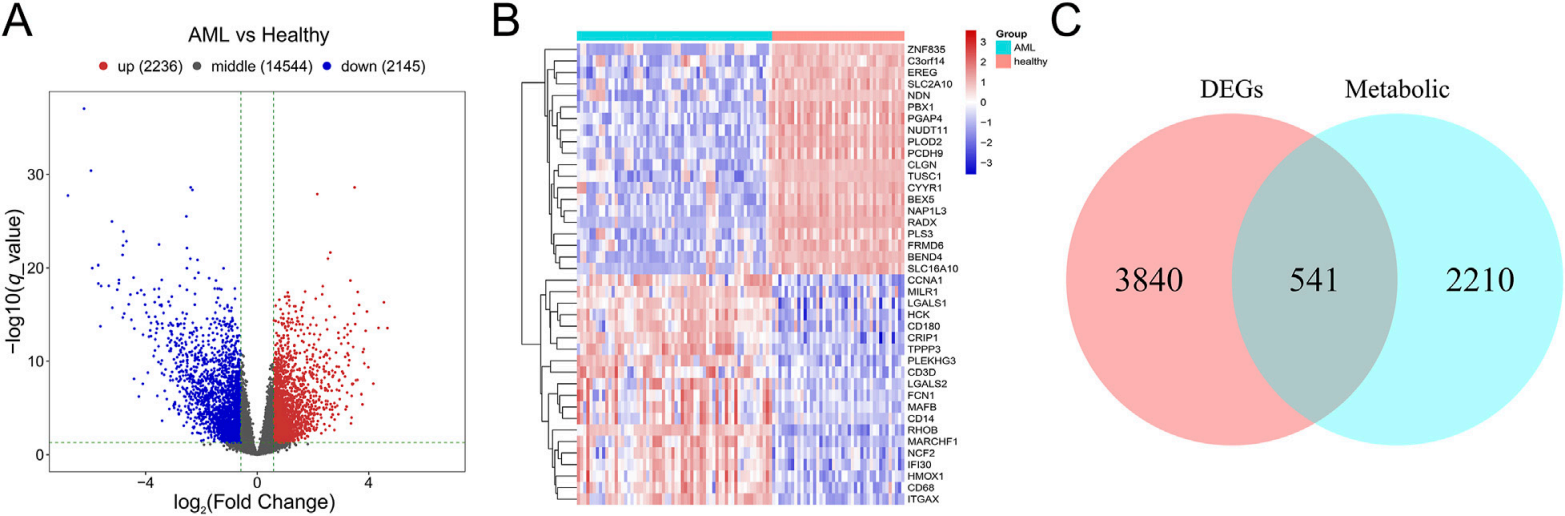
Identification of mDEGs in AML. **(A)** Volcano plot of differentially expressed genes between AML patients and the healthy individuals in GSE63270 dataset. **(B)** Heatmaps of the top 20 differentially expressed genes. **(C)** Venn diagram of the DEGs and the metabolism-related genes from KEGG metabolic pathway-related gene sets.

A total of 4,831 DEGs were identified between AML and control group in GSE63270, including 2,236 upregulated genes and 2,145 downregulated genes (Figures 2A, B). Subsequently, 541 mDEGs were found out using Venn intersect function between the DEGs and the 2,751 metabolism-related genes from KEGG metabolic pathway-related gene sets (Figure 2C).

**FIGURE 3:**
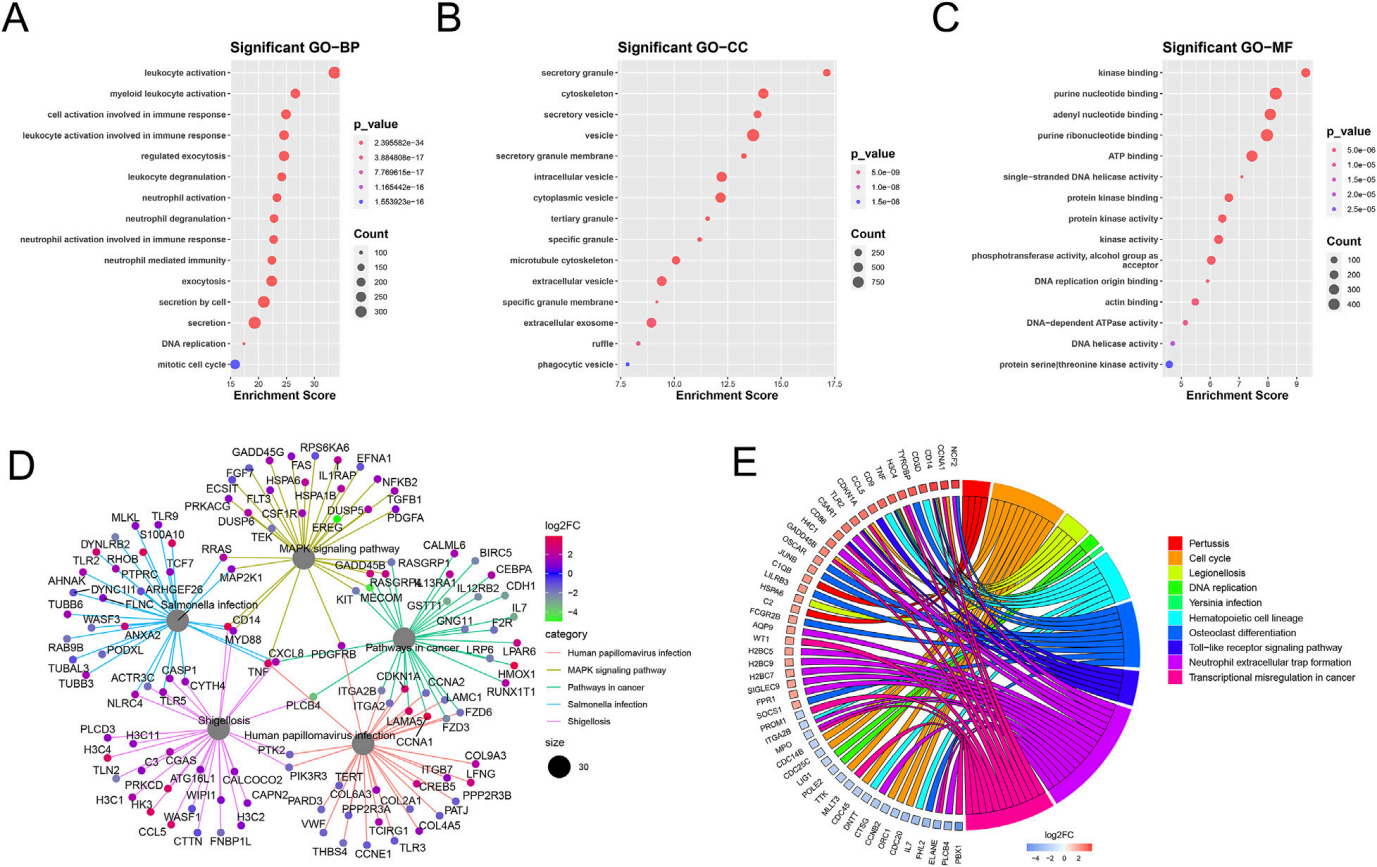
Enrichment analysis of mDEGs. **(A–C)** Bubble plot of the top 15 terms of biological process (BP), Cellular component (CC), Molecular function (MF). **(D)** Cnet plot of KEGG pathways. **(E)** Circle plot of metabolism-related genes KEGG enrichment pathways.

GO and KEGG pathway enrichment analyses of the mDEGs revealed 431 GO terms and 42 pathways (adjusted *p* < 0.05) related to abnormalities in AML, such as myeloid leukocyte activation, transcriptional misregulation in cancer, neutrophil extracellular trap formation, and MAPK signaling pathway (Figure 3).

### 3.2 Prognosis in consensus clustering subgroups of TCGA cohort

**FIGURE 4:**
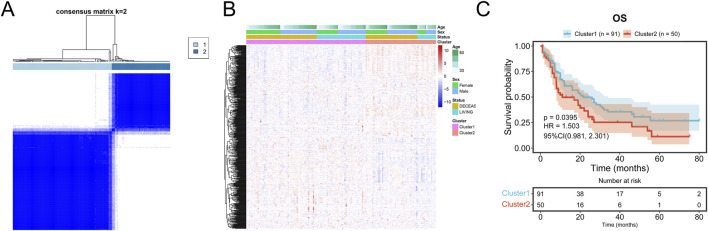
Prognosis in consensus clustering subgroups of TCGA-AML. **(A)** AML patients in TCGA cohort were grouped into two clusters according to the consensus clustering matrix (k = 2). **(B)** Heatmap and the clinical characters of the two clusters classified by the metabolism-related genes in TCGA-AML cohort. **(C)** Kaplan-Meier OS analysis of patients in the Cluster 1 and Cluster 2.

Consensus clustering of AML samples from the TCGA dataset based on the 541 mDEGs revealed two distinct subgroups: Cluster 1 and Cluster 2 (Figure 4A). Cluster 1, characterized by lower metabolic activity (Figure 4B), showed a significantly longer overall survival (OS) compared to Cluster 2 (*p* = 0.0395, Figure 4C), suggesting that different metabolic subgroups may be associated with divergent prognosis in AML patients.

### 3.3 Construction of the metabolism-related risk model for AML prognosis

**FIGURE 5:**
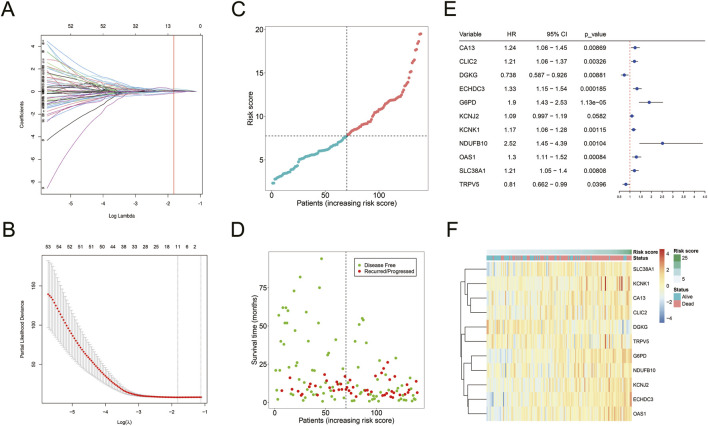
Construction of mDEGs-based prognostic model. **(A)** The coefficients of LASSO cross-validation regression. **(B)** LASSO calculated variable to filter lambda and calculate the minimum lambda and lambda1se. **(C)** The distribution and median value of the risk scores. **(D)** The distributions of OS status in each patient. **(E)** The expression of 11 mDEGs in TCGA-AML cohort. **(F)** Heatmap showing the expression levels of 11 mDEGs.

Univariate Cox regression analysis identified 131 mDEGs associated with AML prognosis. LASSO regression further selected 11 mDEGs for the construction of a prognostic risk model (Figures 5A–D). The model-based risk score was calculated as follows:

Risk score = (CA13 * 0.0093) + (CLIC2 * 0.0248) + (DGKG * −0.1392) + (ECHDC3 * 0.1135) + (G6PD * 0.2315) + (KCNJ2 * 0.0676) + (KCNK1 * 0.0506) + (NDUFB10 * 0.1066) + (OAS1 * 0.0284) + (SLC38A1 * 0.0941) + (TRPV5 * −0.0360).

The model-based risk score was calculated using the expression levels and corresponding risk coefficients of these mDEGs (Figure 5E). It showed that nine genes (CA13, CLIC2, ECHDC3, G6PD, KCNJ2, KCNK1, NDUFB10, OAS1, SLC38A1) had hazard ratio (HR) > 1, categorized as risk genes, while two genes (Fan et al., 2014; Gravina et al., 2023; Li et al., 2019; Liu et al., 2015; Poulain et al., 2017; Sun et al., 2023; Ueno et al., 2019) (DGKG, TRPV5) with HR < 1 were identified as protective genes. A heatmap revealed that high-risk patients had elevated expression of risk genes, while low-risk patients had increased expression of protective genes (Figure 5F).

### 3.4 Validation of the metabolism-related risk score for AML prognosis

**FIGURE 6:**
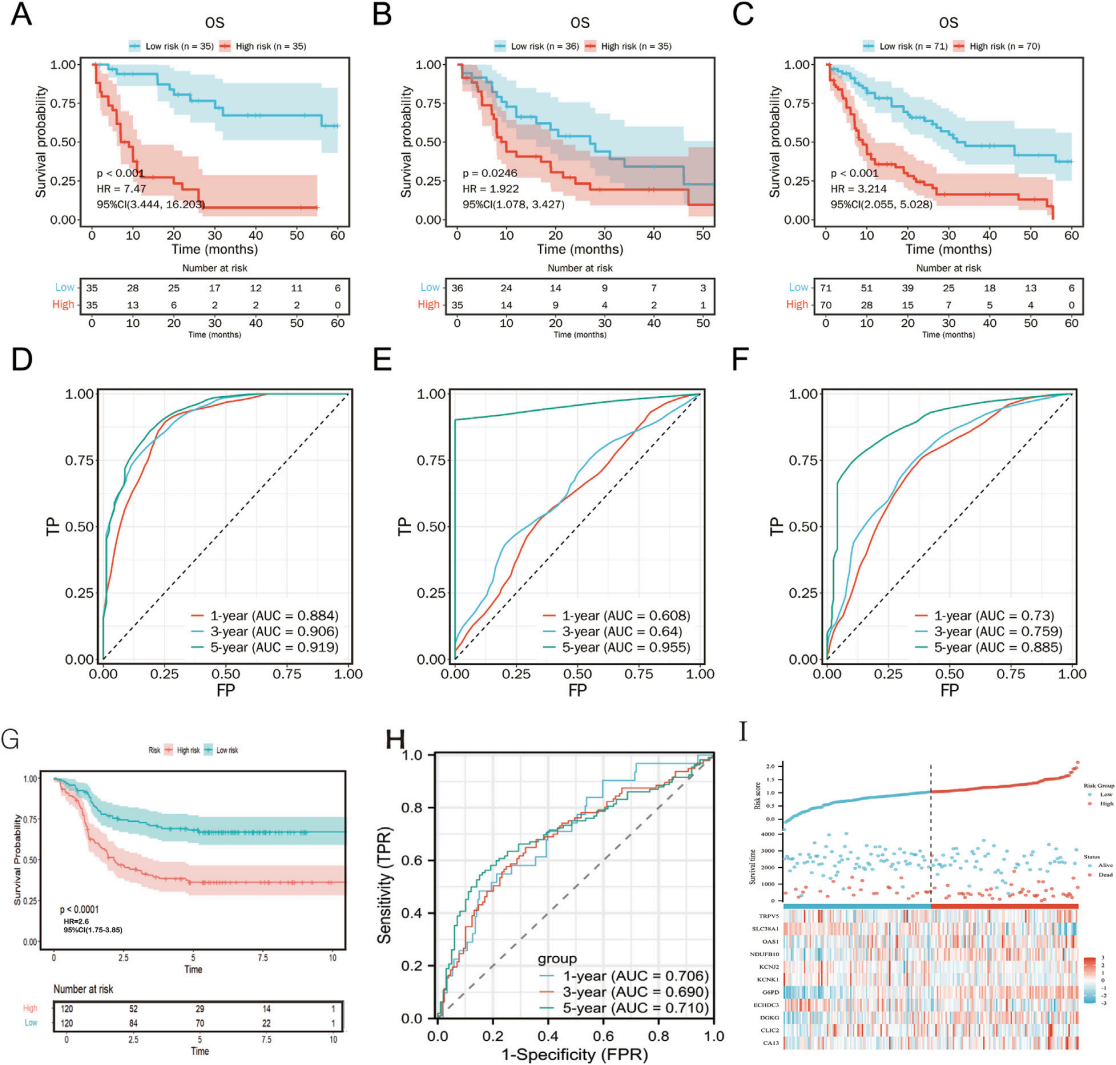
Kaplan-Meier plot and ROC curve for validation of the metabolism-related risk score. Kaplan–Meier survival curves between two risk groups based on the metabolic risk score classification in the training **(A)**, validation **(B)** and the total **(C)** cohort. ROC curve of the metabolic risk score AML prognosis prediction in the training **(D)**, validation **(E)** and the total **(F)** cohort. TP, true positive; FP, false positive. **(G-I)** External validation of TARGET cohort.

The prognostic value of the risk score was validated on TCGA training, validation and total cohorts (Figures 6A–C). Patients were stratified into high- and low-risk groups based on the median risk score. The risk score effectively predicted overall survival (OS) across different time points. High-risk patients exhibited significantly shorter OS compared to the low-risk group (*p* < 0.001, Figure 6F).

In an independent TARGET cohort, the model maintained robust prognostic performance (Figures 6G, H), further confirming its predictive accuracy across diverse patient populations. Expression patterns of the 11 metabolism-related genes were concordant with the risk stratification (Figure 6I).

Compared to the Metabolic Risk Prognostic Signature Index (MRPSI) developed by Wang et al. using 28 genes, our 11-gene model demonstrated superior 5-year OS prediction in both the TCGA (AUC = 0.885 vs. 0.697) and TARGET (AUC = 0.71 vs. 0.688) datasets. While MRPSI employed a support vector machine algorithm, our LASSO Cox regression approach effectively captured prognostic metabolic signals.

### 3.5 Independent prognostic value of the metabolism-related risk score for AML

**FIGURE 7:**
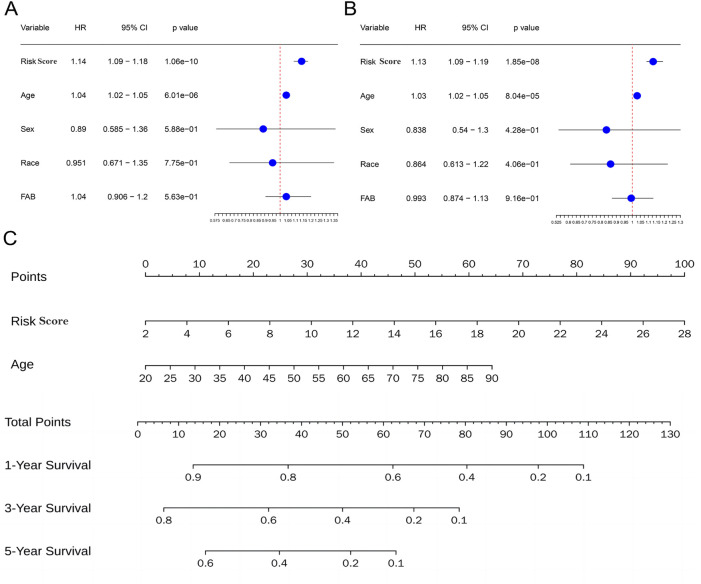
Independent prognostic value of the metabolism-related risk score. **(A)** Univariate analysis in the TCGA cohort. **(B)** Multivariate analysis in the TCGA cohort. **(C)** Construction of a nomogram to predict survival of patients based on clinical parameters and risk score in the TCGA cohort.

To assess the independence of risk score in clinical application, univariate and multivariate Cox regression analyses were performed in TCGA-AML cohort. The risk scores and clinicopathological characteristics, including age, gender, race, andFrench-American-British classification systems (FAB)classification were used as covariates. The results revealed that both age and risk score were independent unfavorable prognostic factors of OS, and risk score is superior to age (Figures 7A, B). Subsequently, a nomogram for OS prediction of AML (1, 3 and 5 years) was established to visualize the metabolism-related risk score (Figure 7C).

### 3.6 Survival and functional analyses of the risk genes

**FIGURE 8:**
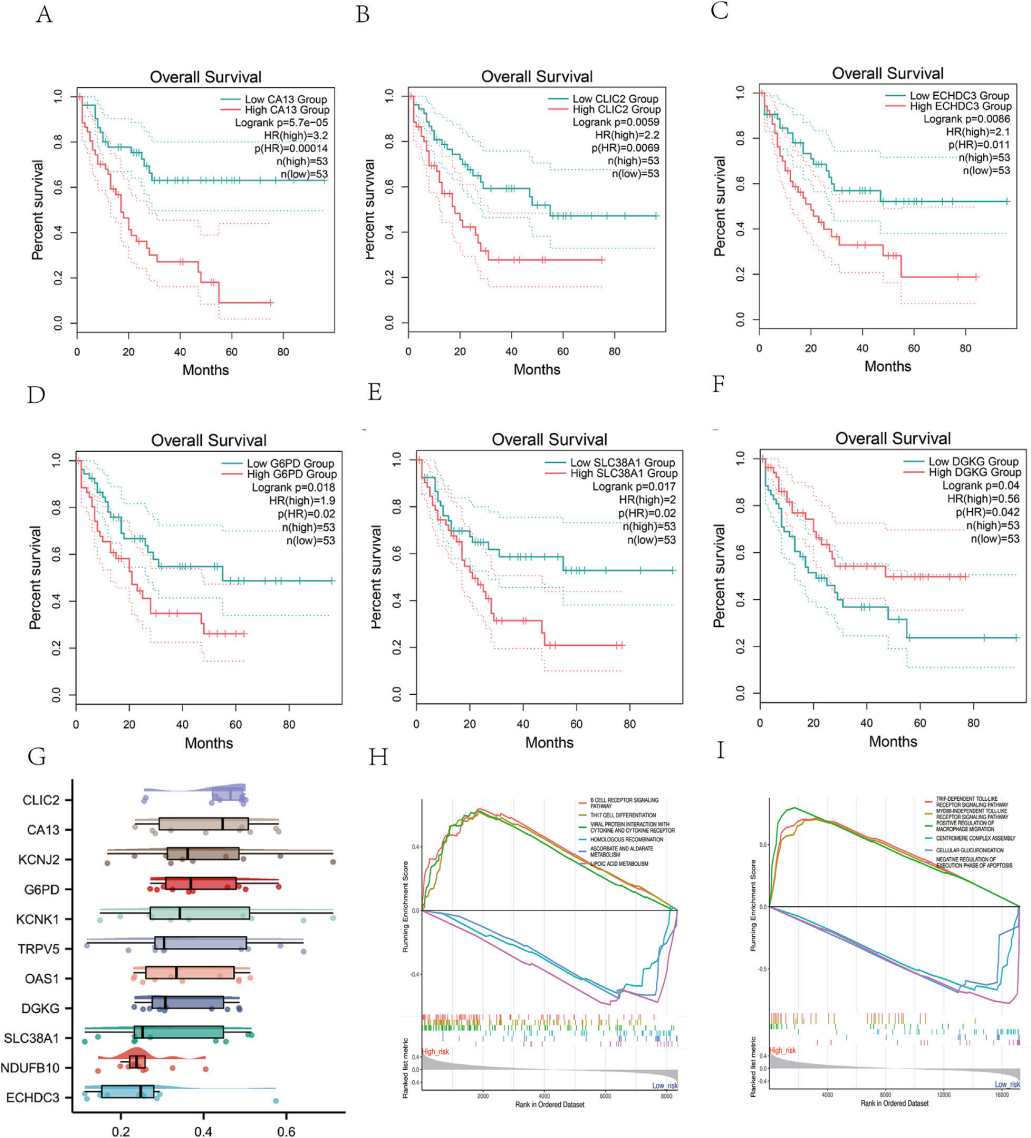
Survival and Functional Analyses of the risk genes. **(A-F)** The Kaplan-Meier curves of AML patients between high and low expression of the genes from metabolism-related risk model. **(G)** Identification of the hub genes from the Friends analysis. The horizontal axis represents the correlation strength, and the vertical axis indicates the gene name.(H).GSEA analysis of KEGG pathway and **(I)** GO biological process.

GEPIA2 database analysis showed that high CA13 expression was significantly correlated with poor OS in AML (HR = 3.2, *p* = 0.00014; Figures 8A–F). Functional interaction analysis identified CLIC2, CA13, and KCNJ2 as potential hub genes in AML prognosis (Figure 8G). GSEA revealed that the risk model predominantly modulates immune and metabolic pathways,.The high-risk phenotype was positively correlated with the B cell receptor signaling pathway and negatively correlated with ascorbate, alternate metabolism, cysteine, and methionine metabolism (Figures 8H, I). These findings suggest that the risk model captures key metabolic alterations in AML that may contribute to disease progression and prognosis.

### 3.7 Immune infiltration and immune checkpoint expression profiles

**FIGURE 9:**
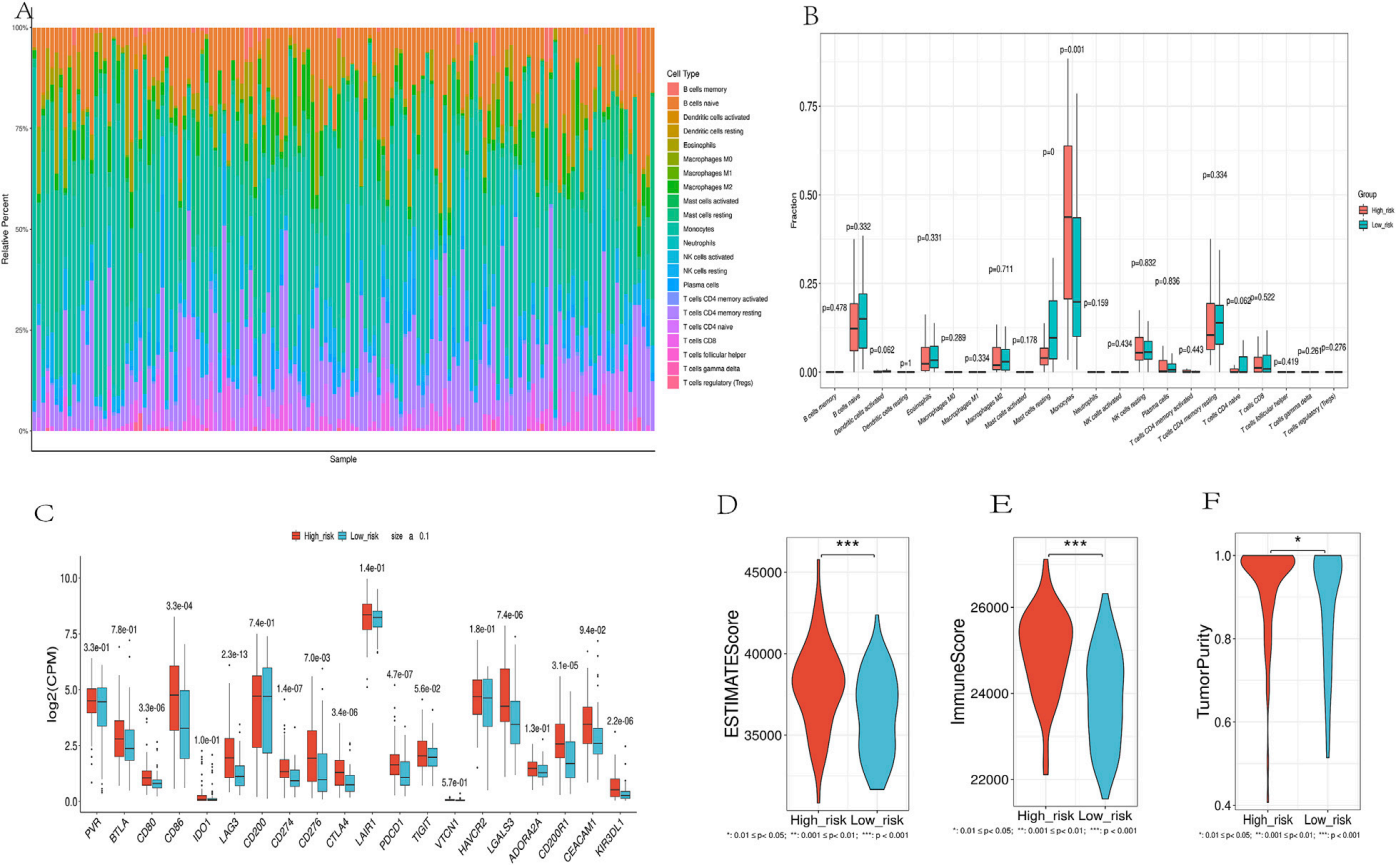
Immune Landscape Analysis. **(A)**. Using the CIBERSORT algorithm, which quantitatively evaluated 22 immune cell types. **(B)**.Immune Cell Distribution Across Risk Groups. **(C)**. Differential expression of immune checkpoint genes between high-risk and low-risk groups. **(D-F)**. Comparison of ESTIMATES score, Immune score, and Stromal score in high-risk and low-risk groups.

CIBERSORT analysis showed a significant correlation between risk scores and the abundance of 22 immune cell types (Figure 9A). High-risk patients had a higher abundance of monocytes, while low-risk patients had more B cell naive and resting mast cells (Figure 9B). Nine immune checkpoint-related genes (CD80, CD86, LAG3, CD274, CTLA4, PDCD1, LGALS3, CD200R1, and KIR3DL1) were significantly upregulated in the high-risk group (Figure 9C), suggesting that high-risk patients may be more suitable for immune checkpoint inhibitor therapy.ESTIMATE analysis of TCGA cancer samples revealed significant differences between high-risk and low-risk patient groups. Patients in the high-risk group had notably higher ESTIMATE scores, Immune scores, and Tumor purity compared to the low-risk group (Figures 9D–F). This suggests a more dense infiltration of stromal and immune cells within the tumor microenvironment of the high-risk patients, as well as a more pronounced presence of immune cells in their tumors.

### 3.8 Drug sensitivity

**FIGURE 10:**
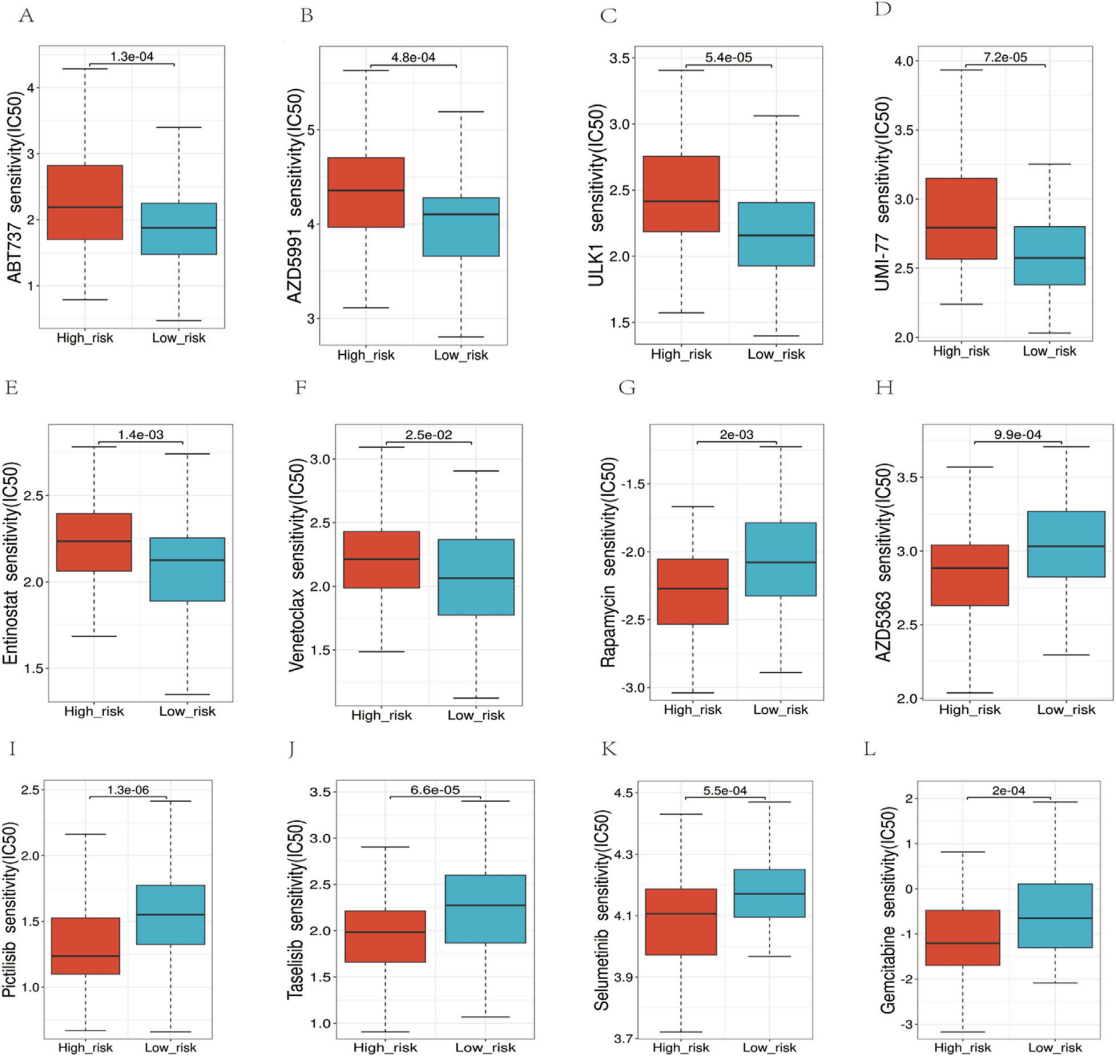
Analysis of the sensitivity to multiple chemotherapy drugs. **(A–F)** In the high-risk group, the IC50 values for ABT737, AZD5991, ULK1, UMI-77, Entinostat, and Venetoclax, were higher. **(G–L)** The IC50 values for Rapamycin, AZD5383, Pictilisib, Taselisib, Selumetinib, Gemcitabine were higher in the low-risk group.

We employed the half-maximal inhibitory concentration (IC50) as a measure to assess the sensitivity of the medication (Figure 10). The results indicated that the high-risk group exhibited significantly higher IC50 values, implying decreased sensitivity to the drugs compared to the low-risk group (*p* < 0.05). Specifically, we observed the following median IC50 values (in μM) for high-risk vs. low-risk groups:ABT737: 8.2 vs. 5.1 (*p* = 0.003),AZD5991: 12.4 vs. 7.8 (*p* = 0.001),ULK1 inhibitor: 6.7 vs. 4.3 (*p* = 0.008),UMI-77: 9.5 vs. 6.2 (*p* = 0.002),Entinostat: 7.9 vs. 5.6 (*p* = 0.004),Venetoclax: 11.3 vs. 7.1 (*p* < 0.001). These results demonstrate that high-risk patients consistently required higher drug concentrations to achieve 50% inhibition of cellular response, indicating reduced drug sensitivity. The most pronounced difference was observed with Venetoclax, where high-risk patients showed a 59% higher IC50 value compared to low-risk patients.

### 3.9 *CA13* correlated with chemoresistance and cell proliferation

**FIGURE 11:**
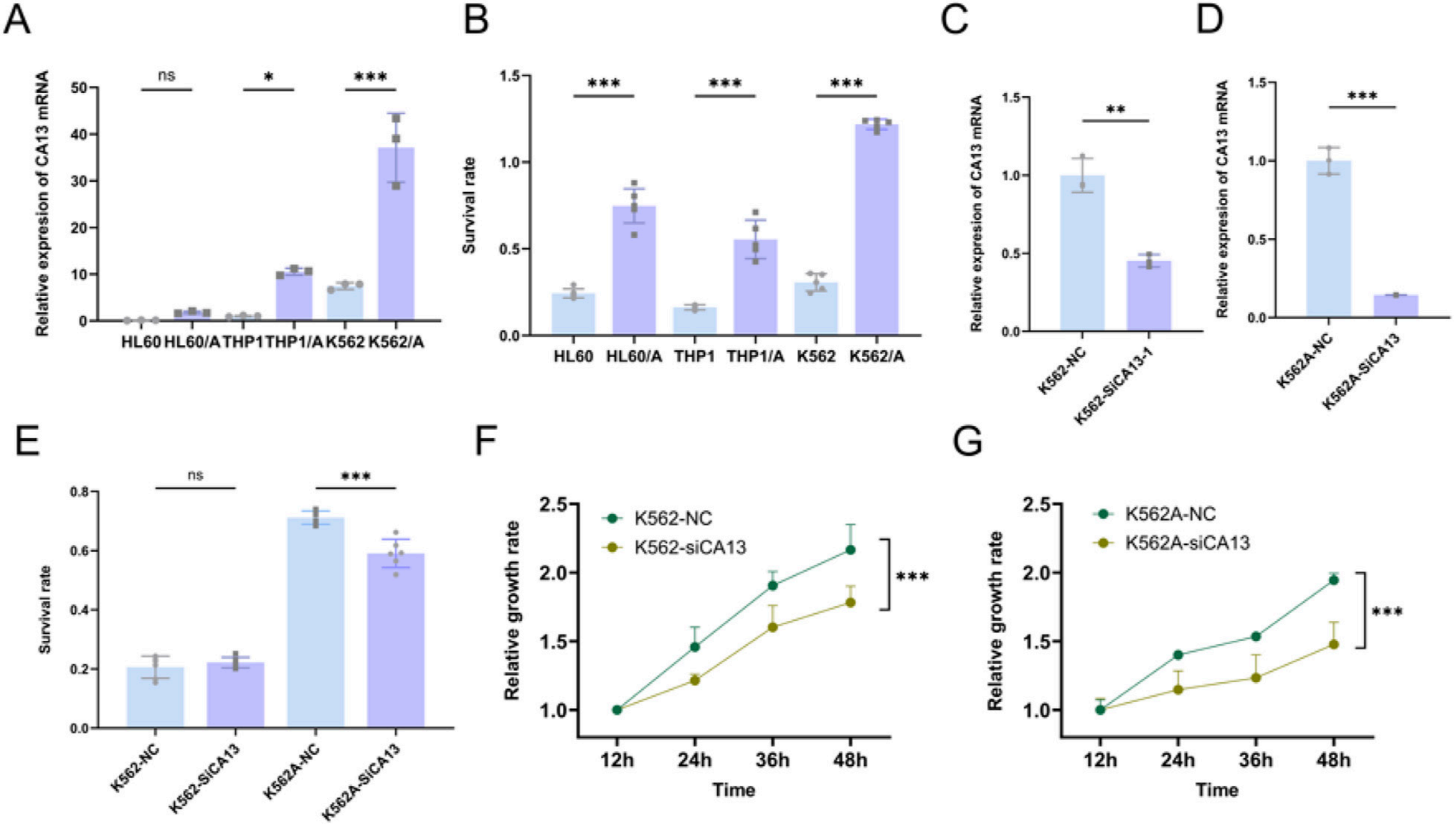
*CA13* correlated with chemoresistance and cell proliferation. **(A)** Relative expression levels of *CA13* in leukemia cell lines (HL60, THP1 and K562), and DOX-resistant cell lines (HL60/A, THP1/A and K562/A). **(B)** Survival rate of leukemia cell lines (HL60, THP1 and K562), and DOX-resistant cell lines (HL60/A, THP1/A, and K562/A) under the treatment of 10 μmol/L DOX for 48 h. **(C–D)** Knocking down *CA13* by siRNA in K562 and K562/A. **(E)** Survival rate under DOX treatment after *CA13* knock-down in K562 and K562/A. **(F–G)** Cell growth curve of K562 and K562/A after *CA13* knock-down. NC: cell line transfected with negative control siRNA. si-CA13: cell line transfected with mixed siRNAs targeting different sites of *CA13*. ***: *p* < 0.001, **: *p* < 0.01, *: *p* < 0.05, ns: *p* > 0.05.

*CA13* showed higher expression levels in DOX (Doxorubicin)-resistant leukemia cell lines (HL60/A, THP1/A and K562/A) compared to their corresponding wild-type cells (HL60, THP1 and K562). Significant difference in survival rates was observed between the DOX-resistant and wild-type leukemia cells after treatment of 10 μmol/L DOX (Figures 11A, B). Then, we knocked down the expression of the *CA13* through siRNA (Figures 11C, D). As shown in (Figures 11F, G), *CA13* knock-down resulted in diminished proliferation of K562 and K562/A cells compared to the control, moreover, a significantly declined survival rate in K562/A cells was found in comparison with that in K562 cells in the milieu of DOX.

## 4 Discussion

This study aimed to enhance the prognostic evaluation of acute myeloid leukemia (AML) by identifying and validating metabolism-related gene signatures. Hundreds of metabolism-related differentially expressed genes (mDEGs) were screened from the GSE63270 cohort, and consensus clustering was performed on TCGA-AML patient data. Significant differences in AML outcomes among the subgroups suggested that mDEGs expressions could be useful for prognosis evaluation. A metabolism-related risk model comprising 11 mDEGs was developed through Cox combined with LASSO regression analysis, and its predictive capacity was validated using KM survival curves and ROC curve analysis.

Our results align with previous findings that metabolic reprogramming plays a critical role in AML progression, drug resistance, and adverse outcomes (Wojcicki et al., 2020; Mishra et al., 2023; Rattigan et al., 2023). In comparison, our model demonstrated AUC values of 0.73 for 1-year OS, 0.759 for 3-year OS, and 0.885 for 5-year OS in the TCGA validation cohort, indicating superior predictive performance, particularly for long-term survival predictions (Wang et al., 2013). The metabolism-related risk score developed in this study has significant potential in clinical settings. It can serve as an independent prognostic factor to supplement existing AML risk stratification systems (Döhner et al., 2017; Döhner et al., 2022).

Gene set enrichment analysis (GSEA) revealed that our risk model predominantly modulates immune and metabolic pathways, indicating that key metabolic alterations contribute to AML progression and prognosis (Ashburner et al., 2000; Kanehisa and Goto, 2000). The correlation of the risk score with immune cell abundance and immune checkpoint-related gene expression further supports the link between metabolism and immune response in AML (Chen et al., 2018).

The study supports the theory that metabolic reprogramming is integral to cancer cell survival and proliferation (Supuran and Winum, 2015). In evaluating targeted therapy efficacy in high-risk AML, we analyzed sensitivity to drugs targeting key pathways using IC50 (Figure 10). For ABT737 (*p* = 0.003), high Bcl-xL expression may reduce binding affinity (Mérino et al., 2012; Parry et al., 2021). Venetoclax, a selective Bcl-2 inhibitor, showed the highest IC50 increase (59%) potentially due to altered mitochondrial outer membrane permeability blocking apoptosis. Our risk model unveiled glycolysis and mitochondrial metabolism activation in the high-risk group, explaining reduced sensitivity to metabolic enzyme inhibitors like ULK1/UMI-77 (Mishra et al., 2023). Resistance to apoptosis-targeting drugs like Venetoclax may stem from sustained pathway activation, suggesting metabolic reprogramming contributes to therapy resistance. Modulating metabolism could improve treatment responses, particularly for metabolically dysregulated high-risk patients. This analysis clarified the reprogramming landscape and resistance mechanisms, guiding personalized approaches. Additionally, our findings suggest that high-risk patients may benefit from metabolism-targeted therapies, which could be integrated into chemotherapy regimens to enhance treatment efficacy, particularly in relapsed or refractory AML patients (Chen et al., 2014; Lee et al., 2018).

Several potential mechanisms by which *CA13* might contribute to chemotherapy resistance were identified based on its known functions. *CA13* is involved in cellular pH regulation, carbon dioxide transport, and cell homeostasis. Abnormalities in these processes can affect the tumor microenvironment, influencing cancer cell survival, proliferation, and resistance to therapy (Angeli et al., 2020; Supuran, 2008). Our *in vitro* experiments showed that decreased expression of CA13 conferred partial sensitivity to chemotherapy in drug-resistant strains. After knock-down of *CA13*, the cell growth rate of drug-resistant strains was significantly reduced compared with wild-type strains, indicating that *CA13* might augment cellular resistance to chemotherapy by regulating the proliferation process of leukemia cells.

There are several limitations to this study. Firstly, the risk model needs validation with larger-scale cohorts to ensure its robustness. Secondly, while we identified *CA13* as a prospective therapeutic target, the underlying mechanisms of *CA13* in regulating drug resistance and its potential as a therapeutic sensitization target in AML require further research. Addressing these limitations is crucial to ensure the robustness and applicability of our findings.

Future research should focus on validating the risk model with larger and more diverse cohorts to confirm its predictive power and applicability. Investigating the detailed mechanisms of *CA13* in drug resistance will be crucial for developing targeted therapies (Yogosawa et al., 2021). Moreover, exploring the potential of combining metabolism-related signatures with other biomarkers could lead to more comprehensive and personalized prognostic models for AML (Long et al., 2020; Zhao et al., 2022). Prospective clinical studies to validate our model and explore its integration into existing risk stratification systems or its use in treatment decision-making would also be valuable (Montesinos et al., 2022).

In summary, our study provides a robust metabolism-related gene signature for prognostic evaluation in AML, highlighting the significance of metabolic pathways in disease progression and treatment resistance. The metabolism-related risk score developed in this study has significant potential in clinical settings, both as an independent prognostic factor and as a guide for therapy strategies (Pastorekova et al., 2006; Supuran and Winum, 2015). Further validation and exploration of targeted therapies based on these findings could significantly improve AML patient outcomes.

## 5 Conclusion

Based on bioinformatics data analysis extracted from the public database, we constructed a novel metabolism-related gene signature for AML, which could be an effective assistant for the risk stratification and outcome prediction in AML. In addition, *CA13* was verified to be a poor prognosis factor and potential therapeutic target due to its regulation of drug resistance, providing a new perspective on AML chemotherapy and deserved meticulous investigation.

## Data Availability

The original contributions presented in the study are included in the article/Supplementary Material, further inquiries can be directed to the corresponding author.
