# Supplementary material for: Prognostic implications of metabolism-related genes in acute myeloid leukemia
Source: Front Genet. 2024 Oct 3;15:1424365. doi: 10.3389/fgene.2024.1424365 (PMC11484252; doi:10.3389/fgene.2024.1424365)
Supplement: Supplementary file 1 [file Table3.DOCX]

| Item | Sequence |
| --- | --- |
| CA13 Forward Primer | 5’- GCG AGC ACA ACG GTC CTA TT -3’ |
| CA13 Reverse Primer | 5’- TGG TCG GAG GGA AGA GTC AT - 3’ |
| siRNA CA13-Homo-401 | Sense: 5'-GCA CAA CGG UCC UAU UCA CTT-3' |
|  | Antisense: 5'-GUG AAU AGG ACC GUU GUG CTT-3' |
| siRNA CA13-Homo-585 | Sense: 5'-GAU GAC ACA GAG AAC AAA UTT-3' |
|  | Antisense: 5'-AUU UGU UCU CUG UGU CAU CTT-3' |
| siRNA CA13-Homo-774 | Sense: 5'-GCA GCU CAU GAA CCA GAU GTT-3' |
|  | Antisense: 5'-CAU CUG GUU CAU GAG CUG CTT-3' |

Supplyment table 1
